# Matched Field Processing Based on Least Squares with a Small Aperture Hydrophone Array

**DOI:** 10.3390/s17010071

**Published:** 2016-12-30

**Authors:** Qi Wang, Yingmin Wang, Guolei Zhu

**Affiliations:** School of Marine Science and Technology, Northwestern Polytechinal University, Xi’an 710072, China; qwang208@nwpu.edu.cn (Q.W.); ywang@nwpu.edu.cn (Y.W.)

**Keywords:** matched field processing, small aperture, passive localization, least squares, acoustic fields

## Abstract

The receiver hydrophone array is the signal front-end and plays an important role in matched field processing, which usually covers the whole water column from the sea surface to the bottom. Such a large aperture array is very difficult to realize. To solve this problem, an approach called matched field processing based on least squares with a small aperture hydrophone array is proposed, which decomposes the received acoustic fields into depth function matrix and amplitudes of the normal modes at the beginning. Then all the mode amplitudes are estimated using the least squares in the sense of minimum norm, and the amplitudes estimated are used to recalculate the received acoustic fields of the small aperture array, which means the recalculated ones contain more environmental information. In the end, lots of numerical experiments with three small aperture arrays are processed in the classical shallow water, and the performance of matched field passive localization is evaluated. The results show that the proposed method can make the recalculated fields contain more acoustic information of the source, and the performance of matched field passive localization with small aperture array is improved, so the proposed algorithm is proved to be effective.

## 1. Introduction

Matched field processing (MFP) is a kind of generalized beamforming which is widely used in the oceanic geometric and geoparameters inversion, underwater passive localization, noise restriction, virtual time reversal mirror, and so on [1,2,3,4]. However, the application of MFP is seriously restricted due to its sensitivity to environmental mismatch caused by the water depth, sound speed profile, sediment parameters, ship noise, etc. [5,6,7,8,9]. In addition, the MFP usually needs to sample the acoustic fields sufficiently; thus, the receiver array must have enough hydrophones which can cover the whole water column [10,11]. As a result, even in the shallow water, the array is still about one hundred meters, and is readily influenced by the wind and ocean current. So the application of MFP is severely hindered by this factor.

Hamson and Wilson have done some research on the effect of different layouts of the receiver arrays to MFP [5,12]. In their works, the received fields of vertical linear array at each frequency can be expressed as a sum of individual modal contributions with the normal mode propagation model, and it is shown that the small aperture vertical array can only adequately sample the high order normal mode, and it is inadequate for the low order mode which contributes more. Further theoretical studies have shown [13] that the peak width of MFP decreases with the number of modes used, and the maximum width is approximately equal to the interference distance between the highest order mode and the lowest order modes. When the modes used increase, the localization resolution of range and depth become more accurate. In order to improve the localization resolution, the receiver array must can sample all the modes, so the vertical linear array must have enough hydrophones which can cover the whole water column.

In this paper, a new method called matched field processing based on least squares (LSMFP) with small aperture hydrophone array is proposed. It can localize the underwater acoustic source using a small aperture array without require lots of hydrophones.

The remainder of this paper is organized as follows. Section 2 describes the theoretical analyses of acoustic fields using the normal mode propagation model, and constructs the proposed matched field processing based on least squares in the sense of minimum norm. Section 3 presents lots of numerical experiments in the shallow water illustrating the performance of LSMFP in comparison to conventional MFP (CMFP) results, where the receiver arrays are some small aperture vertical linear ones. Section 4 summarizes the findings and conclusions drawn from this work.

## 2. Least Squares MFP Model Formulation

### 2.1. Acoustic Fields with Normal Mode

In the stratified shallow water like pekeris waveguide, the acoustic field can be described as follows [5]:

(1) $$p\left(r,z\right)=\sum_{m=1}^{M}p_{m}\left(r,z\right)$$

where,

(2) $$p_{m}\left(r,z\right)=\frac{je^{-j\pi /4}}{\rho \left(z_{s}\right)\sqrt{8\pi }}\frac{\varphi_{m}\left(z\right)\varphi_{m}\left(z_{s}\right)e^{j\xi_{m}r}}{\sqrt{\xi_{m}r}}$$

According to Equation (1), the fields at each frequency can be expressed as a sum of individual modal contributions where *p(r,z)* is the acoustic pressure field at the location *(r,z)*, and *ρ* is the density of the water, $z_{s}$ is the depth of source, $\varphi_{m}$ is the mode depth function of the *m*th mode, $\xi_{m}$ is the horizontal wave number of the *m*th mode, *M* is the number of normal mode.

Derived from Equation (1), the acoustic field can be written simply as:

(3) $$p\left(r,z\right)=\sum_{m=1}^{M}d_{m}\left(r,z_{s}\right)\varphi_{m}\left(z\right)$$

where $d_{m}\left(r,z_{s}\right)$ is the mode amplitude of the *m*th mode:

(4) $$d_{m}\left(r,z_{s}\right)=\frac{je^{-j\pi /4}}{\rho \left(z_{s}\right)\sqrt{8\pi \xi_{m}r}}\varphi_{m}\left(z_{s}\right)e^{j\varphi_{m}r}$$

For a receiver array of *N* hydrophones which covers the whole water column, assuming that the depth of *n*th hydrophone is $z_{n}$ , the fields of all the receivers as Equation (3) can be expressed as follows [12]:

(5) $$p_{N}={\left[\begin{array}{ccc} p(r,z_{1}) & \cdots & p(r,z_{N}) \end{array}\right]}^{T}$$

Substitute Equation (3) to Equation (5), the fields $p_{N}$ can be written as:

(6) $$p_{N}=\left[\begin{array}{ccc} \varphi_{1}\left(z_{1}\right) & \cdots & \varphi_{M}\left(z_{1}\right)\\ \vdots & \ddots & \vdots \\ \varphi_{1}\left(z_{N}\right) & \cdots & \varphi_{M}\left(z_{N}\right) \end{array}\right]\cdot \left[\begin{array}{c} d_{1}\\ \vdots \\ d_{M} \end{array}\right]$$

And $p_{N}$ can be described by a matrix as follows:

(7) $$p_{N}=\varphi_{NM}d_{M}$$

where $p_{N}$ represents the pressure fields of *N* hydrophones; $\varphi_{NM}$ is the sample matrix of mode depth function, and the dimension is N×M; $d_{M}$ is the mode amplitudes vector, and its dimension is M×1. When the environmental parameters are well known, $\varphi_{NM}$ can be obtained accurately by the normal mode without requiring any other information. In this paper, we use the KRAKEN normal mode program [14].

In this way, the received acoustic fields are decomposed into depth function and amplitudes of modes. In particular, we found that no matter whichever receiver arrays are adopted, the amplitudes of normal mode in a range-independent ocean waveguide are all the same. If we can derive the mode amplitudes from the received fields, let ${\hat{d}}_{M}$ be the estimation, then the received acoustic field of any array can be reconstructed as follows:

(8) $${\hat{p}}_{N}=\varphi_{NM}{\hat{d}}_{M}$$

Assuming a vertical linear array which can sample all the modes sufficiently needs at least *N* hydrophones, now only *L* elements of this array near the water surface can work well. So the array becomes a small aperture vertical linear array, and the received fields of this small array can be expressed as:

(9) $$p_{L}=\varphi_{L}d_{M}$$

Assuming we can get the estimation of $d_{M}$ from the received fields of small array in Equation (7), then the fields of N−L remainder hydrophones can be written as:

(10) $$p_{N-L}=\varphi_{N-L}{\hat{d}}_{M}$$

where ${\hat{d}}_{M}$ is also the estimation of $d_{M}$. As we known, $p_{L}$ and $p_{N-L}$ are subsets of $p_{N}$, $\varphi_{L}$ and $\varphi_{N-L}$ are subsets of $\varphi_{N}$, so the fields of the whole water derived from the small aperture array can be described as:

(11) $$p_{N}={\left[\begin{array}{cc} p_{L}^{T} & p_{N-L}^{T} \end{array}\right]}^{T}$$

Substitute Equation (10) into Equation (11), we can get that:

(12) $$p_{N}={\left[\begin{array}{cc} p_{L}^{T} & \varphi_{N-L}\cdot {\hat{d}}_{M} \end{array}\right]}^{T}$$

Now the problem that we consider is how to obtain the estimation of mode amplitudes $d_{M}$ from the fields of the small array. When *L* is larger than *M*, the mode amplitudes $d_{M}$ can be estimated by computing the pseudo inverse directly using the least squares [12]:

(13) $${\hat{d}}_{M}={\left(\varphi_{LM}^{H}\varphi_{LM}\right)}^{-1}\varphi_{LM}^{H}p_{N}$$

This method of computing the mode amplitudes works well for overdetermined systems. However, when L<M, Equation (9) is underdetermined and the least square does not work, some new method must be adopted to obtain the estimation of mode amplitudes.

### 2.2. Estimation of Mode Amplitudes by Least Squares

According the Nyquist sample theorem, a small aperture array with *L* elements can only sample *L* modes at the most, so the mode amplitudes estimated from Equation (7) can only include *L* elements which have contribution to the fields. Additionally, the sample matrix of mode depth function φ that is used must have some errors, because there is a mismatch between the model and real environment, meanwhile, there are also some system errors and noise. Therefore, the real received fields of the small array can be expressed as follows:

(14) $$p_{L}+\Delta p_{L}=\left(\varphi_{L}+\Delta \varphi_{L}\right)d$$

where $p_{L}$ is the received fields without errors, $\varphi_{L}$ is the mode depth function without errors, $\Delta p_{L}$ and Δφ are the perturbations which represent all the errors and mismatch.

For estimating the mode amplitudes accurately, the errors or perturbations must be as small as possible, and the optimum problem can be described like this:

(15) $$\begin{array}{c} \min_{\Delta \varphi_{L},\Delta P_{L},d}{∥\left[\Delta \varphi_{L},\Delta P_{L}\right]∥}_{2}^{2},\\ s.t.,p_{L}+\Delta p_{L}=\left(\varphi_{L}+\Delta \varphi_{L}\right)d \end{array}$$

where ${∥\cdot ∥}_{2}$ denotes the 2-norm, and sometimes the constraint condition can be written as $p_{L}+\Delta p_{L}\in Range\left(\varphi_{L}+\Delta \varphi_{L}\right)$. For simplification, Equation (14) is expressed as:

(16) (B+D)x=0

Here, $B=[\varphi_{L},p_{L}]$ is the data augmented matrix, $D=[\Delta \varphi_{L},\Delta P_{L}]$ is the augmented matrix correction or the perturbation matrix. It is obvious that whether B or D is L×M+1 matrix, $x={\left[d^{T},-1\right]}^{T}$ is (M+1)×1 vector. The problem that Equation (15) described is to find a perturbation matrix $D\in C^{L\times M+1}$ whose norm square is minimized, which involves B+D in rank deficiency. So the optimization problem of Equation (15) can be written to be a standard least squares form:

(17) $$\min{∥Bx∥}_{2}^{2},s.t.x^{H}x=2$$

Now, we decompose the augmented matrix B by the singular value decomposition (SVD) method:

(18) $$B=U{\Sigma V}^{H}$$

where $\Sigma =diag\left(\sigma_{1},\sigma_{2},\cdots ,\sigma_{M+1}\right),\sigma_{1}\geq \sigma_{2}\geq \cdots \geq \sigma_{M+1}$ are the singular values, $v_{1},v_{2},\cdots v_{M+1}$ are the right singular vectors, and they all have specific physical meanings in our problem: the former *M* singular vectors correspond to the *M* modes of acoustic fields, and the last one is least square solution, usually it is called total least square solution [15,16,17,18]

(19) $$\hat{d}=\frac{1}{v(1,M+1)}\left[\begin{array}{c} v(2,M+1)\\ \vdots \\ v(M+1,M+1) \end{array}\right]$$

where v(i,M+1) is the *i*th element of the (M+1)th column in the right singular matrix V.

However, as previously shown, an array with *L* elements can only sample *L* modes at the most. Assuming a small array with *L* hydrophones can sample *k* modes completely, so we can only know that *k* singular values satisfying $\sigma_{1}\geq \sigma_{2}\geq \cdots \geq \sigma_{k+1}$, in this sense the meaning of singular values and vectors have changed, and Equation (19) is not the optimum total least square solution.

Let $v_{i}$ be a vector in the subspace S defined by:

(20) $$S=Span\{v_{k+1},v_{k+2},\cdots ,v_{M+1}\}$$

Then every right singular vector corresponds to a total least square solution:

(21) $$x_{i}=y_{i}/\alpha_{i},i=k+1,k+2,\cdots ,M+1$$

where $\alpha_{i}$ is the first element of $v_{i}$, and $y_{i}$ consists of the other elements of $v_{i}$, that is $v_{i}={\left[\alpha ,y_{i}^{T}\right]}^{T}$. So there are M+1−k TLS solutions, we must find an only TLS solution in some special meaning. Golub and Vanloan proposed a TLS solution in the sense of minimum norm [19], which can solve this problem perfectly. Their algorithm is summarized as follows:

- Decompose the augmented matrix B by SVD: $B={U\Sigma V}^{H}$.
- Let *L* is the elements of small array, *M* is the mode number in a shallow waveguide, *k* is the number of main singular value. If *L* is less than *M*, then let *k* equal to *L*, else let *k* equal to *M*.
- Let $V_{1}=v_{k+1},v_{k+2},\cdots ,v_{M+1}$, it is obviously that $V_{1}$ consists of the latter M+1−k column of V, so the dimension of $V_{1}$ is (M+1)×(M+1−k).
- Let ${\bar{v}}_{1}$ denotes the first row of $V_{1}$, $V_{2}$ consists of the last rows of $V_{1}$, viz. $V_{1}=\left[{\bar{v}}_{1};V_{2}\right]$, so the dimension of $V_{2}$ is M×(M+1−k), and $V_{2}$ is a row less than $V_{1}$. Then the TLS solution in the sense of minimum norm can be expressed as:
  (22) $$\hat{d}=\frac{V_{1}{\bar{v}}_{1}^{H}}{{\bar{v}}_{1}{\bar{v}}_{1}^{H}}=\alpha^{-1}V_{1}{\bar{v}}_{1}^{H}$$

As previously mentioned, we can only sample *k* modes completely; however, given the solution estimated in Equation (22) suppose that the number of modes is equal to the columns of φ in Equation (14), then it is consistent with the physical scene.

### 2.3. Matched Field Processing Based on Least Squares

The conventional matched field processor (CMFP) is usually also called the Bartlett processor, and its ambiguity surface is as follows [4,20]

(23) $$B_{CMFP}\left(r,z\right)=\omega_{c}^{H}\left(r,z\right)R\left(r_{s},z_{s}\right)\omega_{c}\left(r,z\right)$$

(24) $$\omega_{c}\left(r,z\right)=\frac{p_{c}\left(r,z\right)}{\left|p_{c}^{H}\left(r,z\right)\right|}$$

where R is the cross spectral density matrix of received fields:

(25) $$R\left(r_{s},z_{s}\right)=\frac{1}{K}\sum_{k=1}^{K}p\left(r_{s},z_{s}\right)p^{H}\left(r_{s},z_{s}\right)$$

In Equation (24) $p_{c}\left(r,z\right)$, which are called replica fields, are the acoustic fields on the assumption that the virtual source is located at (r,z) by the normal mode or other propagation model, $\omega_{c}$ is the normalized weight vector. In Equation (25) $p(r_{s},z_{s})$, which are called the measured fields, are the received acoustic fields of the real source at $\left(r_{s},z_{s}\right)$. The essence of MFP is to obtain the square of correlation coefficient between measured fields and replica forecasted by propagation models. The dimensions of $p_{c}\left(r,z\right)$, $\omega_{c}\left(r,z\right)$, and $p(r_{s},z_{s})$ are all L×1 for small array with *L* elements.

In this paper, one method in which the acoustic fields received by small array are used directly to match with replica is called CMFP, and the other method in which the fields used is recalculated with Equation (12) is called the least squares matched field processor (LSMFP):

(26) $$B_{LSMFP}\left(r,z\right)=\omega_{cls}^{H}\left(r,z\right)R_{cls}\left(r_{s},z_{s}\right)\omega_{cls}\left(r,z\right)$$

(27) $$\omega_{cls}\left(r,z\right)=\frac{p_{cls}\left(r,z\right)}{\left|p_{cls}^{H}\left(r,z\right)\right|}$$

(28) $$R_{cls}\left(r_{s},z_{s}\right)=\frac{1}{K}\sum_{k=1}^{K}p_{cls}\left(r_{s},z_{s}\right)p_{cls}^{H}\left(r_{s},z_{s}\right)$$

where $p_{cls}\left(r,z\right)$ is also the replica acoustic fields on the assumption that the virtual source is located at (r,z), $\omega_{cls}\left(r,z\right)$ is the normalized weight vector, $p_{cls}\left(r_{s},z_{s}\right)$ is the reconstructed acoustic fields using the received fields of small array by LS at the location $\left(r_{s},z_{s}\right)$ for real source. In Equation (27), the dimensions of $p_{cls}\left(r,z\right)$, $\omega_{cls}\left(r,z\right)$, and $p_{r}\left(r_{s},z_{s}\right)$ are all N×1 even that the receiver array is a small vertical linear array with *L* elements.

Comparing Equations (23) and (26), we can see that the performance of the two methods is depends on the similarity between the received or reconstructed received fields and the replica fields. To evaluate the similarity between the reconstructed ones and the real pressure fields, the generalized cosine-squared between two vectors is defined as:

(29) $$\cos^{2}\left(p_{N},{\hat{p}}_{N}\right)=\frac{\left(p_{N}^{H}{\hat{p}}_{N}\right)\left({\hat{p}}_{N}^{H}p_{N}\right)}{\left(p_{N}^{H}p_{N}\right)\left(\hat{p_{N}^{H}}\hat{p_{N}}\right)}$$

When the whole acoustic fields are reconstructed, the performance of matched field processing or other algorithm with small aperture array is improved.

## 3. Numerical Experiments and Performance Analysis

### 3.1. Environmental Model

**Figure 1:**
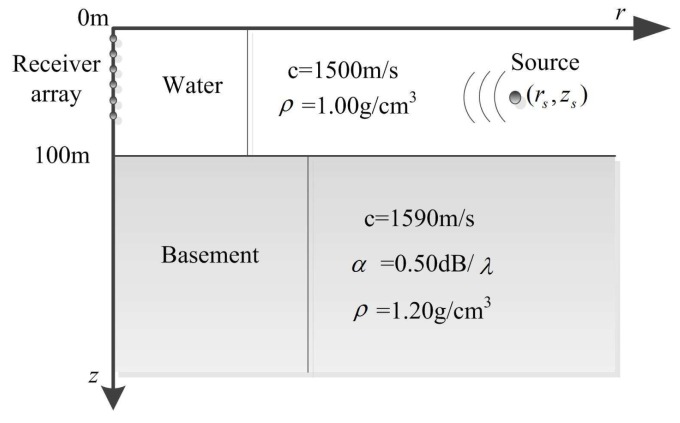
The typical calibration case (CALIB) environmental model in the workshop. It is a two-layer environmental model with geoacoustic parameters of water and basement.

To address the problem of matched field processing in shallow water, a workshop was held in May 1993 at the Naval Research Laboratory where some simulated data and cases were provided to the community of users to test the algorithms. A particular cases from the workshop was the calibration case (CALIB) where the environmental scenario was the typical Pekeris shallow water shown as Figure 1. The water depth is about 100 m, the water surface is pressure released, and the bottom or basement is an infinite liquid space. Both of the sound speed profiles are isovelocity.

According to the normal mode propagation model, in shallow water like Figure 1 the highest order mode number is 22. For sampling the acoustic fields without distortion the vertical linear receiver array which spans the whole water column must consist of at least 22 elements equally spaced.

**Figure 2:**
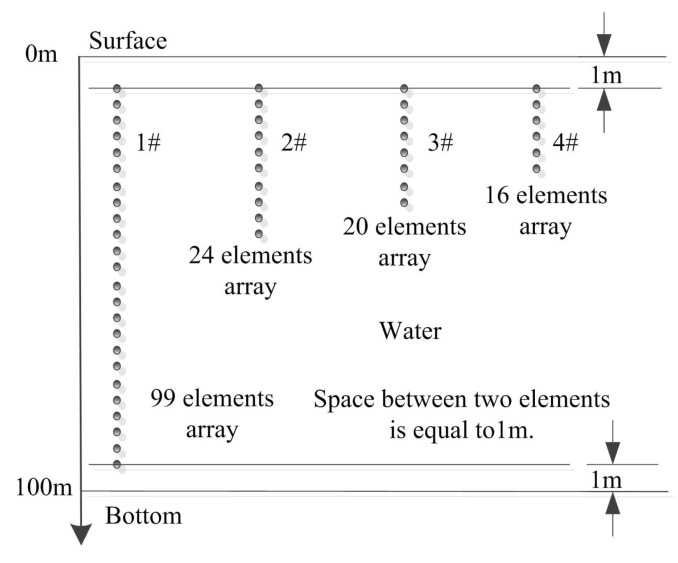
The layout of the receiver arrays. 1# array is a large vertical array, its aperture is 98 m. 2#, 3#, and 4# arrays are all small vertical linear ones, their apertures are 23 m, 19 m and 15 m, respectively.

In order to evaluate the performance of the proposed method, lots of simulations with small vertical arrays which are located near by the water surface are processed in the classical shallow water. Figure 2 is the layout of all the receiver arrays. In particular, 1# array is a large aperture array with 99 hydrophones which covers all the water column, it is used for the reference, 2#, 3# and 4# arrays are small aperture arrays that consist of 24, 20, 16 hydrophones respectively. The first element of all the arrays is at a depth of 1 m, and the interval between two elements is 1m. In addition, the signal is emitted by a transducer at 80 m depth and 7000 m range, the single tone of signal is 500 Hz.

### 3.2. Results of Localization

**Figure 3:**
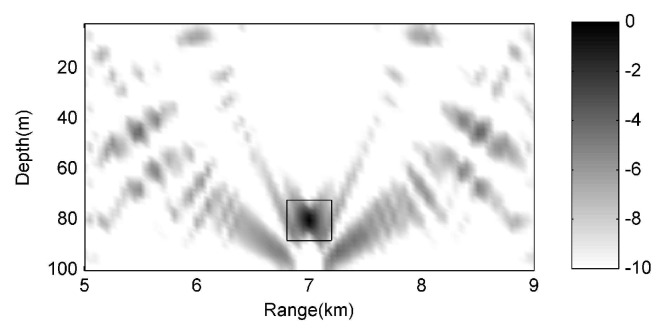
Bartlett ambiguity surface of 1# large array. The target in the rectangle is the localization result, and it is the real acoustic source.

In all the numerical experiments, the signal to noise ratio (SNR) is equal to 20 dB, and the real environment can match the environmental model perfectly. The ambiguity surface obtained using 1# large array for the Bartlett method is shown in Figure 3.

The source location (range, depth) estimated is about (7000 m, 80 m) as in the rectangle, and the localization result is clear and correct, because 1# array can fully span the water column such that the sampling of acoustic fields is adequate for all the normal modes. It can also be seen that the mainlobe and sidelobe are easy to recognize and can not be confused with each other. The signal to interference plus noise ratio (SINR) is approximate 16.65 dB, the peak to background ratio (PBR) is about 10.49 dB, where SINR and PBR are defined as follows:

(30) $$SINR=10lg(\sigma_{S}^{2}/\sigma_{I}^{2})$$

(31) $$PBR=10lg(\sigma_{S}^{2}/\sigma_{0}^{2})$$

In which $\sigma_{S}^{2}$ is the signal power, $\sigma_{0}^{2}$ is the average power of the background, and $\sigma_{I}^{2}$ is the largest sidelobe power plus noise power, which we consider as the interference. The results in Figure 3 show that MFP can work well and the localization can proceed successfully when the fields can be sampled adequately by a large array, and now the generalized cosine-squared of received fields is equal to 1.

**Figure 4:**
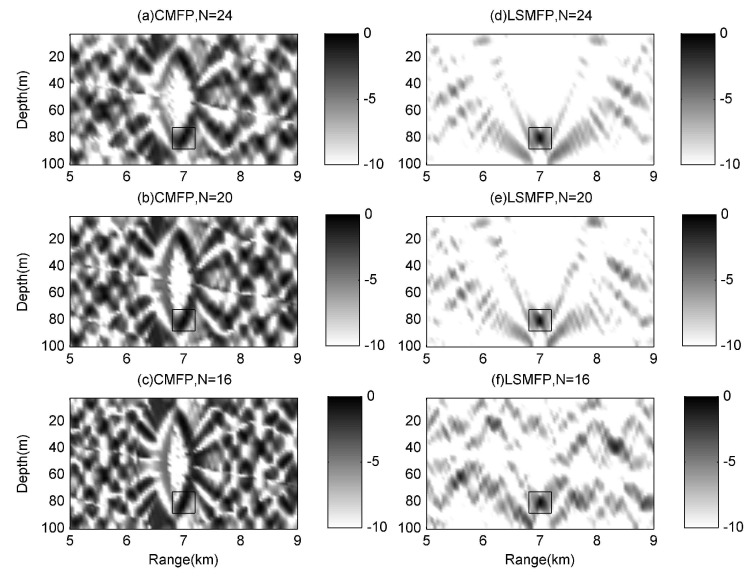
Comparison among ambiguity surfaces of three small arrays. (**a**–**c**) are ambiguity surfaces of 2#, 3#, 4# array for CMFP method; (**d**–**f**) are ambiguity surfaces of 2#, 3#, 4# array for LSMFP method.

As shown in Figure 4, the localization results of all the subfigures are about (7000 m, 80 m) in rectangles. Although all the localizations are processed successfully, we can not distinguish the mainlobe from the background, because the sidelobes are too many, and the amplitudes are close to that of the mainlobe. Even though 2# array has 24 hydrophones, the amplitudes of the mainlobe are only about 0.5 dB more than that of the maximum sidelobe in Figure 4a–c, so the mainlobe is more easily confused with the sidelobes. The localization results in Figure 4d–f are obviously clearer than those in Figure 4a–c. Particularly, in Figure 4d,e, the surfaces look very much like Figure 3, because all the mode amplitudes are estimated successfully by the least squares. We can also find that the more hydrophones the small array consists of, the more approximate with the real mode amplitudes the estimated ones are, when comparing the three surfaces on the right side. Therefore, the performance of LSMFP which uses the estimated mode amplitudes is improved.

It should be noticed that there is some common ground when examining Figure 4a–c, that is, these subfigures are symmetrical with the center, because only parts of fields are obtained by the small aperture array, which leads us to confuse the reflection from the water surface with that from the bottom. In this case, the water surface and the bottom look the same, this is problematic for MFP with small aperture arrays. The proposed LSMFP can perform particularly well in dealing with the confusion caused by sampling deficiently. Although the confusion phenomenon exists in Figure 4f, it is shown that the least squares can address this confusion, but this depends on the number of hydrophones used; the algorithm fails when the receiver hydrophones are too few.

### 3.3. Performance Analysis

Table 1 shows the comparison among the localization results of three small arrays and the large array to analyse the performance of CMFP and LSMFP quantitatively. It is known that 1# large array in which the number of hydrophones is 99 can sample all the modes sufficiently, this layout is ideal, and thus it can be used as reference. For 1# array GCOS is 1.0, SINR and PBR is also maximum no matter which processor is adopted. For three small arrays without using the least squares, the GCOSs are 0.6866, 0.5002 and 0.3247 respectively, viz. that the original received fields of small arrays only have part of acoustic fields information, so SINR and PBR are less than the reference. When using the least squares to reconstruct the received fields of small arrays, the GCOSs become 0.9917, 0.8928 and 0.5887 respectively, according to Table 2. After that the performance of MFP is clearly proved. It can also be seen from Table 1 and Table 2 that the larger GCOS is before using LSMFP, the more accurate the localization is when using MFP.

**Figure 5:**
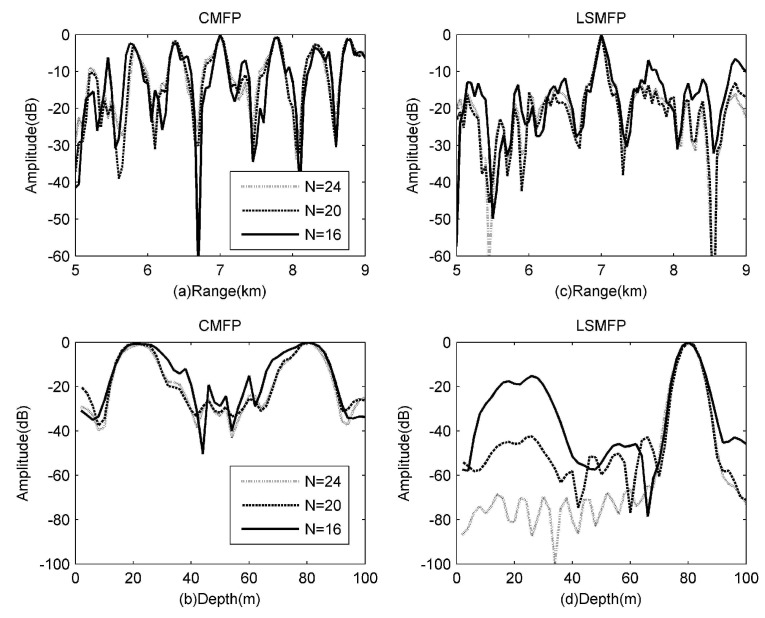
Localization slices of small arrays at the position of (7000 m, 80 m). (**a**,**b**) are the range and depth slices of CMFP; (**c**,**d**) are the range and depth slices of LSMFP.

In Figure 5a,b, it can be seen that the value of mainlobe and that of maximum sidelobe is almost the same, the mainlobe at the range of 7000 m is hard to distinguish with the sidelobe at the range of 7800 m, meanwhile, the mainlobe at the depth of 80 m is also hard to distinguish from the sidelobe at the depth of 22 m. From Figure 5c,d, it can be seen that LSMFP can suppress the sidelobe greatly, especially for the 2# array which consists of 24 hydrophones and for which the sidelobe suppression is the most efficient, e.g., the sidelobe at the range of 7800 m is suppressed by about 10 dB, and the sidelobe at the depth of 22 m is suppressed by about 70 dB. For the 4# array, which consists of 16 hydrophones, the suppression is the worst of all; however, the sidelobe and the mainlobe can also be distinguished from oneanother. Meanwhile, it is apparent that no matter which processor is adopted, the array with more hydrophones performs better localization.

**Figure 6:**
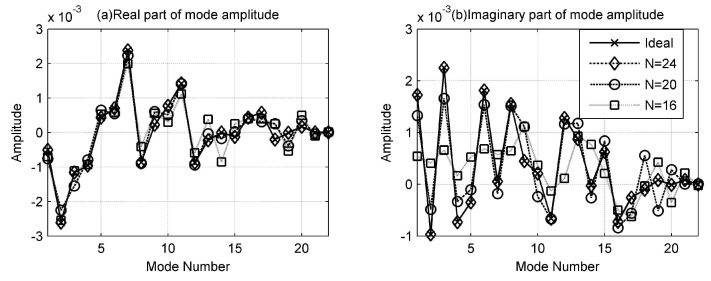
The mode amplitudes estimated by the least squares with small arrays. (**a**,**b**) are the real and imaginary parts of mode amplitudes, respectively.

As previously shown, LSMFP can improve the localization performance of small aperture arrays, because the accuracy of mode amplitudes estimated is improved. In Figure 6, it can be clearly seen that the estimation for real parts is more accurate than that of imaginary parts, the estimation for lower order modes is more accurate than that of higher order modes, and the more elements the small array consists of, the more accuracy the estimated amplitudes are. It can also be seen that when the number of hydrophones is 24, the mode amplitudes estimated are almost the same as the real ones, so the hydrophones of the small arrays is enough when the amount of hydrophones is larger than that of the modes.

## 4. Conclusions

The layout of receiver array is always problematic for matched field processing, and this seriously hinders its application. To address this issue, a method called matched field processing based on least squares with small aperture hydrophone array is proposed. As previously shown, using the least squares method to estimate the mode amplitudes is essential for a successful localization with small arrays. The acoustic fields can be reconstructed when the mode amplitudes are estimated. After that, the source localization performance of MFP is greatly improved.

To evaluate the performance, two approaches are compared by lots of numerical experiments in shallow water. The first of these is CMFP, which uses the received fields directly, and the second is LSMFP which uses the reconstructed fields of small array. The experiments are processed successfully and the results show that, firstly, the least squares can recalculate the received fields of small array successfully, this is the basis of performance improving for source localization. Secondly, LSMFP must use the mode depth function which rests with the environment, so it is also sensitive to the environmental mismatch like other MFP methods. Thirdly, the performance of LSMFP depends on the estimations of mode amplitudes, and the estimations for low order modes are more accurate, because small aperture array is sufficient for low order modes, at this point enough receiver hydrophones is better for LSMFP regardless of any array.

In summary, the main aim of the proposed approach is to shorten the aperture of the receiver array. This is successfully achieved by combining the prior environmental information with received fields sufficiently, so other algorithms based on acoustic fields like virtual time reversal mirror can also be used, and it will be easy to realize.

## Figures and Tables

**Table 1:** Comparison among small and large arrays for conventional matched field processing (CMFP).

Array	Hydrophones	Range (m)	Depth (m)	SINR (dB)	PBR (dB)	GCOS
1#	99	7000	80	16.65	10.49	1.0
2#	24	7000	80	8.60	3.41	0.6866
3#	20	7000	80	8.22	2.80	0.5002
4#	16	7000	80	7.95	2.40	0.3247

**Table 2:** Comparison among small and large arrays for matched field processing based on least squares (LSMFP).

Array	Hydrophones	Range (m)	Depth (m)	SINR (dB)	PBR (dB)	GCOS
1#	99	7000	80	16.65	10.49	1.0
2#	24	7000	80	16.32	10.34	0.9917
3#	20	7000	80	15.78	10.22	0.8928
4#	16	7000	80	13.58	8.12	0.5887
